# A new species of pea crab of the genus *Serenotheres* Ahyong & Ng, 2005 (Crustacea, Brachyura, Pinnotheridae) from the date mussel *Leiosolenus* Carpenter, 1857 (Mollusca, Bivalvia, Mytilidae, Lithophaginae) from the Solomon Islands

**DOI:** 10.3897/zookeys.623.10272

**Published:** 2016-10-11

**Authors:** Peter K. L. Ng, Chistopher Meyer

**Affiliations:** 1 Lee Kong Chian Natural History Museum, Faculty of Science, National University of Singapore, 2 Conservatory Drive, Singapore 117377, Republic of Singapore; 2 Department of Invertebrate Zoology, U.S. National Museum of Natural History, Smithsonian Institution, Washington DC 20013, USA

**Keywords:** Pinnotheridae, taxonomy, symbiotic crab, new species, symbiotic crab, Serenotheres janus, Solomon Islands

## Abstract

The pea crab genus *Serenotheres* Ahyong & Ng, 2005 (Pinnotheridae) is currently only represented by one species, *Serenotheres besutensis* (Serène, 1967). A new species is now assigned to this genus, described from a date mussel *Leiosolenus obesus* Carpenter, 1857 (Mollusca: Bivalvia: Mytilidae: Lithophaginae) collected in the Solomon Islands. *Serenotheres janus* **sp. n.** differs from *Serenotheres besutensis* in possessing a conspicuously broader carapace, with the lateral margins of the dorsal lamellum distinctly produced and the posterolateral part deeply concave, the dorsal lamellum being highest at the median cleft, the rostrum is relatively more prominent, the surfaces of the anterolateral margin and hepatic region are less prominently pitted and eroded, the ischiomerus of the third maxilliped is relatively more rectangular, and the P2 merus is proportionately longer.

## Introduction

Ahyong and Ng (2005) revised the species in the Indo-West Pacific pinnotherid genera *Durckheimia* De Man, 1889 (type species *Durckheimia carinipes* De Man, 1889) and *Xanthasia* White, 1846 (type species *Xanthasia murigera* White, 1846), and established two new genera, namely *Serenotheres* Ahyong & Ng, 2005, for *Durckheimia besutensis* Serène, 1967; and *Tridacnatheres* Ahyong & Ng, 2005, for *Xanthasia whitei* De Man, 1888. They commented that *Serenotheres* differed from all pinnotherid genera not only by the unusual carapace which has an additional large plate above its normal carapace surface (a dorsal lamellum) which overhangs the frontal margin, but also by possessing a two-segmented third maxilliped palp (Ahyong and Ng 2005: 121). It is also the only known pinnotherid associated with the rock-boring bivalves of the mytilid subfamily Lithophagidae (see Schmitt et al. 1973).

In this paper, a new species of *Serenotheres* is described from the Solomon Islands.

## Material and methods

The specimen examined is deposited in the U.S. National Museum for Natural History (USNM), Smithsonian Institution, Washington D.C.

The following abbreviations are used: MXP3 = third maxilliped; P2–P5 = pereiopods 2–5 (first to fourth ambulatory legs), respectively. Measurements (in millimetres) are of the carapace width and length, respectively. The terminology used essentially follows that in Manning (1993) and Ahyong and Ng (2005).

### Molecular data

A mtDNA COI barcode was generated from this individual following standard Sanger sequencing protocols as outlined in Meyer (2003). PCR primers jgLCO1490 and jgHCO2198 (Geller et al. 2013) were used. The resulting sequence is ACCCTTATATTTTATCTTCGGAGCTTGGGCAGGTATAGTAGGAACTTCTTTAAGTTTAATAATTCGAGCTGAACTTAGACAACCAGGCAGACTTATTGGAAATGACCAAATTTATAATGTAATAGTTACAGCCCATGCTTTTGTTATAATTTTCTTTATAGTTATACCAATTATAATCGGAGGCTTCGGAAACTGATTAGTTCCTTTAATACTTGGGGCCCCAGATATAGCATTCCCTCGTATAAACAATATAAGATTTTGACTCTTACCTCCATCTTTATCACTCTTACTTACAAGAAGAATAGTTGAAAGTGGAGTAGGAACAGGATGAACTGTTTATCCTCCTCTAGCTTCAGCTATTGCCCATGCTGGAGCTTCTGTAGATTTAGGAATTTTCTCGCTTCATTTGGCCGGTGTATCGTCAATCTTAGGAGCAGTAAATTTTATTACTACTGTAATTAATATACGATCATATGGAATAATGATAGACCAAATACCACTATTTGTCTGATCAGTATTTATCACCGCAATCCTCCTACTTCTATCCCTACCGGTTCTAGCAGGAGCTATTACCATACTATTAACAGATCGTAATCTAAATACCTCATTCTTTGACCCAGCCGGTGGTGGAGATCCTGTTCTCTATCAACATTTATTT. This record is deposited in Genbank under submission number KX949585.

## Systematics

### Family Pinnotheridae De Haan, 1833 Genus *Serenotheres* Ahyong & Ng, 2005

#### Serenotheres janus sp. n.

Taxon classificationAnimaliaDecapodaPinnotheridae

http://zoobank.org/FD849337-EB57-46F6-965D-8CECBADECD5F

**Figure 1.**
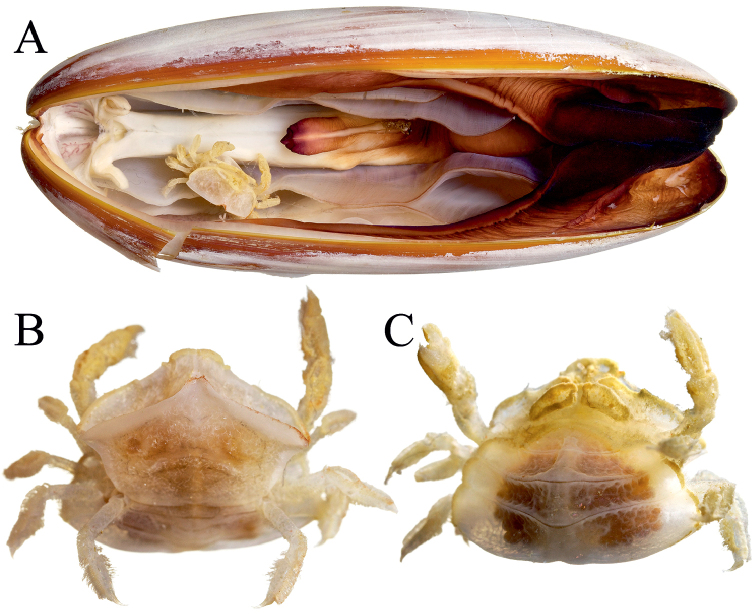
Colour in life. *Serenotheres janus* sp. n., holotype ♀ (8.9 × 7.9 mm) (USNM). **A** in situ in host date mussel, *Leiosolenus obesus* **B** dorsal view **C** ventral view. Photographs courtesy of Zachariah Kobrinsky and David Liittschwager.

**Figure 2.**
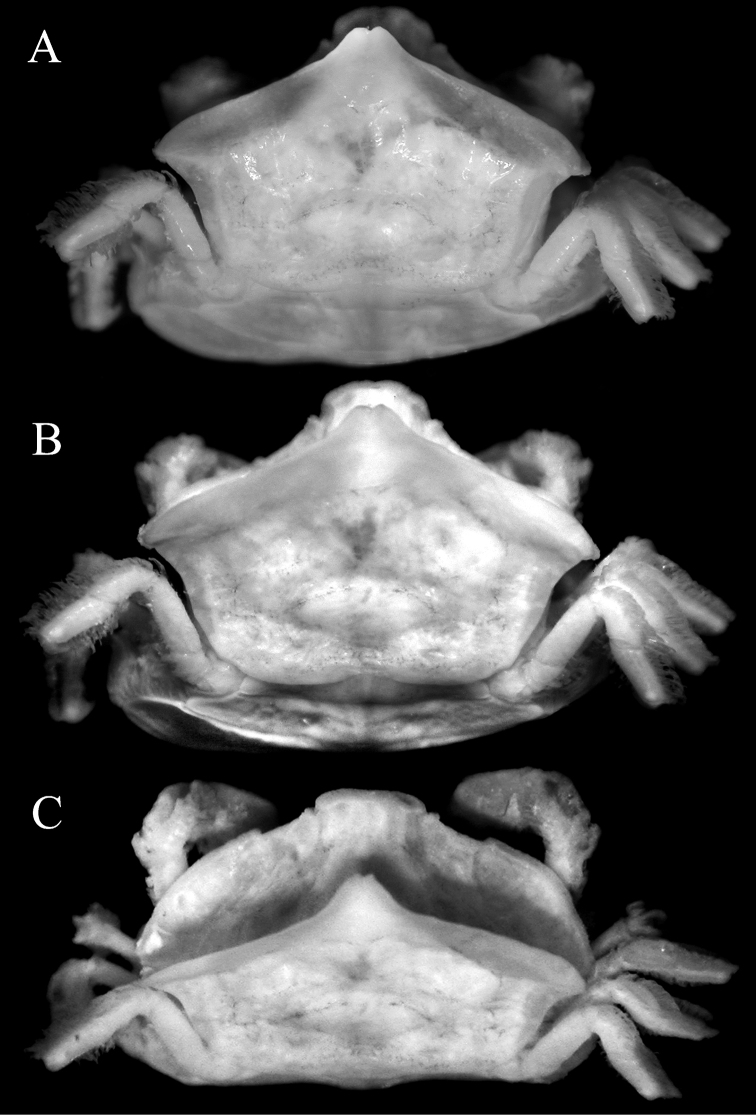
*Serenotheres janus* sp. n., holotype ♀ (8.9 × 7.9 mm) (USNM). Dorsal views of cephalothorax.

**Figure 3.**
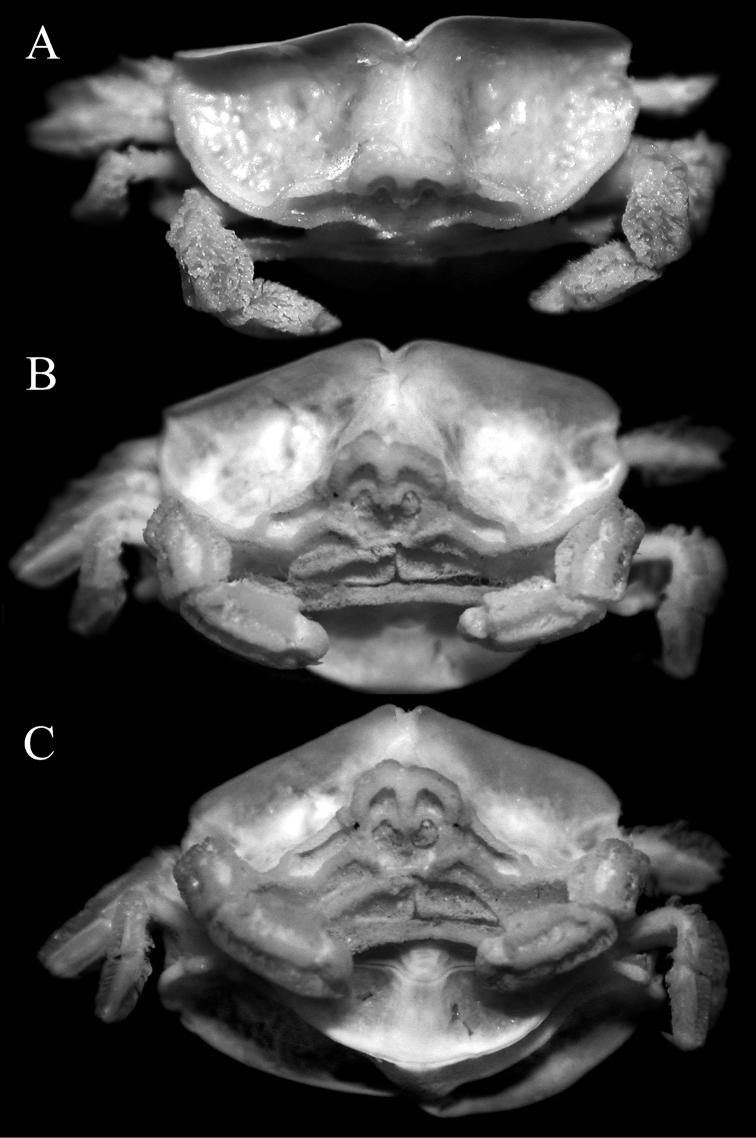
*Serenotheres janus* sp. n., holotype ♀ (8.9 × 7.9 mm) (USNM). Frontal views of cephalothorax.

**Figure 4.**
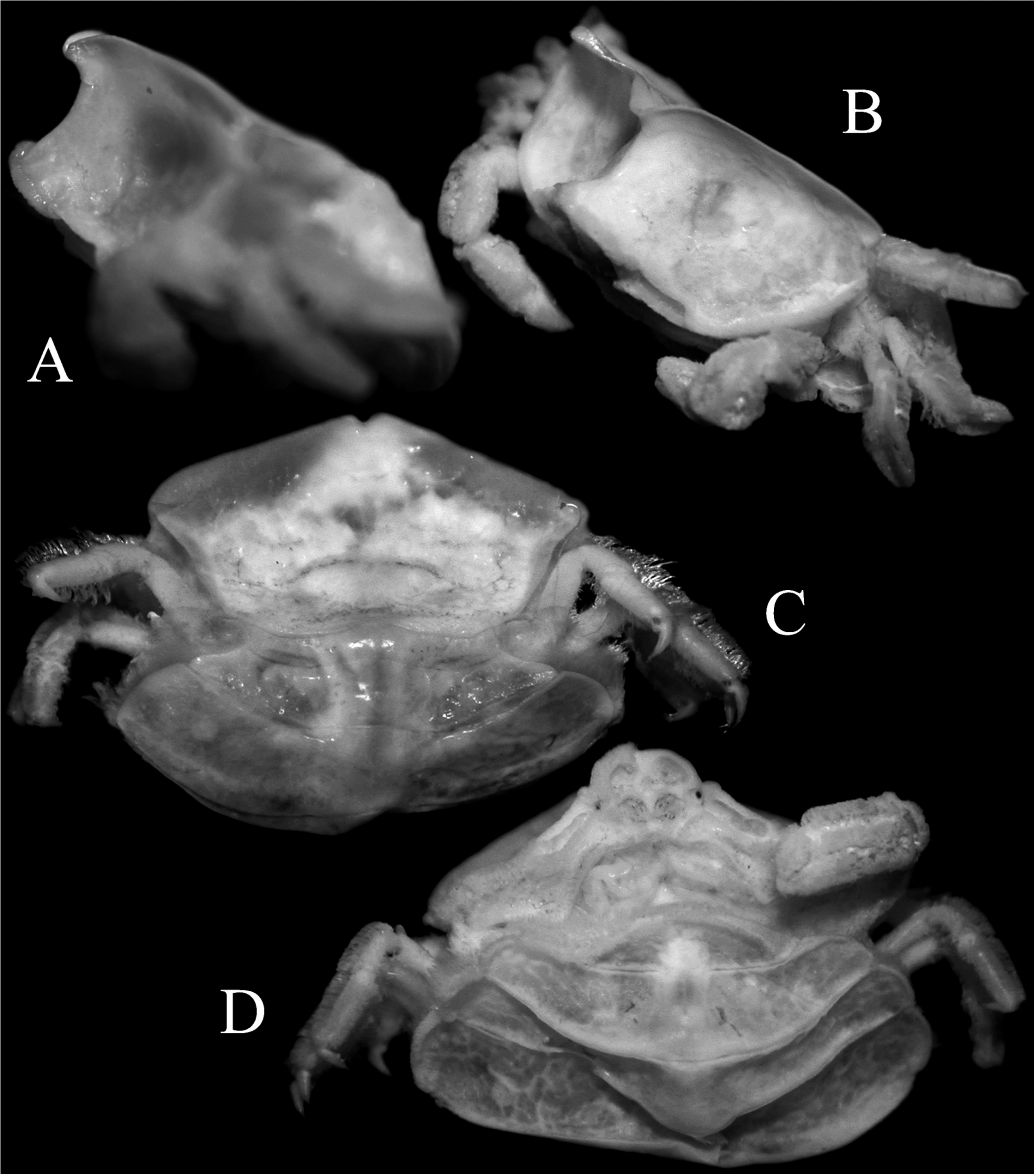
*Serenotheres janus* sp. n., holotype ♀ (8.9 × 7.9 mm) (USNM). **A** angled view of cephalothorax **B** lateral view of cephalothorax **C** posterior part of dorsal lamellum of carapace and abdominal somites 1–3 **D** abdominal somites 5, 6 and telson.

**Figure 5.**
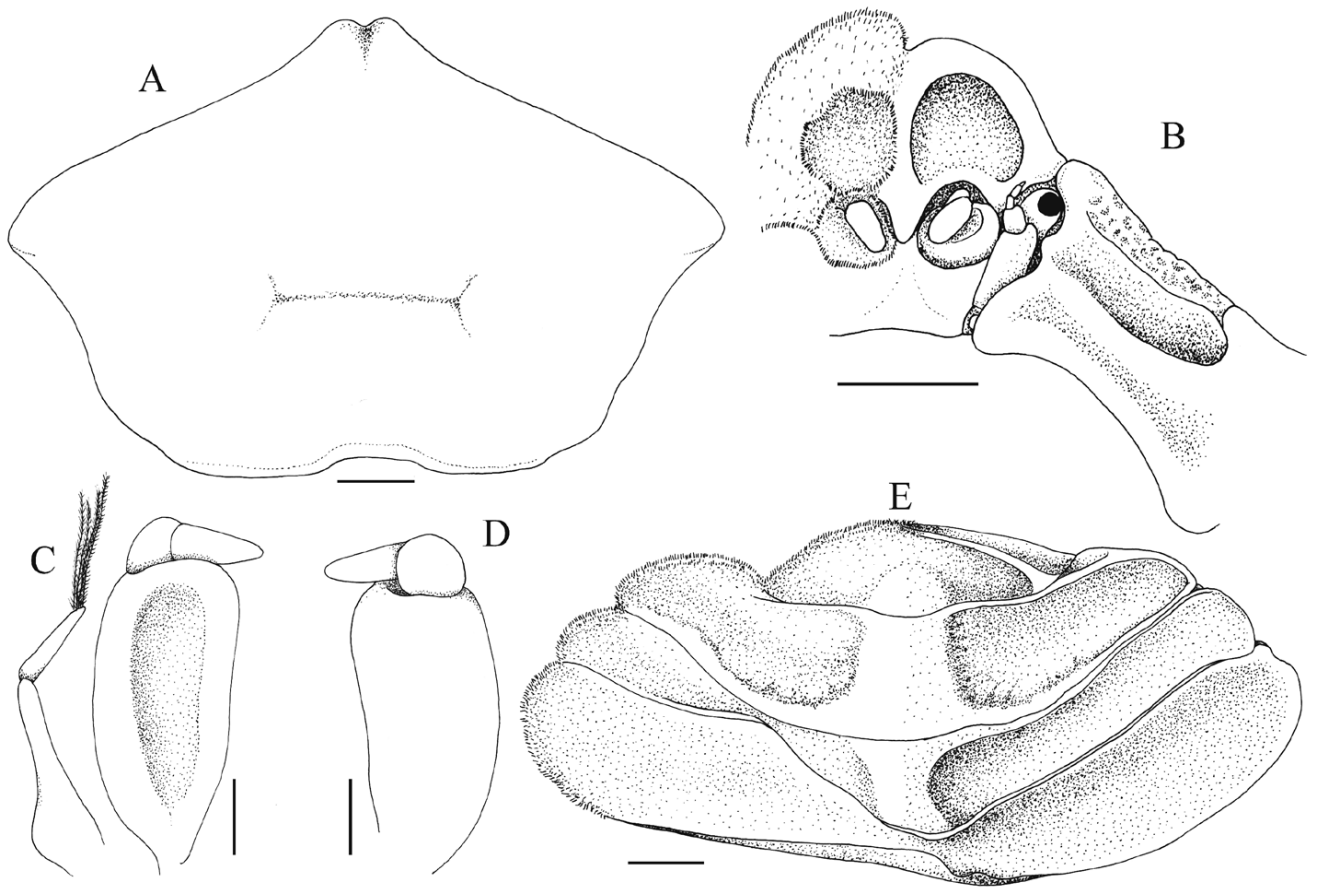
*Serenotheres janus* sp. n., holotype ♀ (8.9 × 7.9 mm) (USNM). **A** overall carapace dorsal lamellum **B** frontal view of cephalothorax (left side denuded) **C** outer view of right MXP3 and exopod (denuded) **D** inner view of right MXP3 (denuded) **E** abdominal somites 4–6 and telson (pits and eroded areas not drawn, left side denuded). Scales: **A, B, E** = 1.0 mm; **C, D** = 0.5 mm.

**Figure 6.**
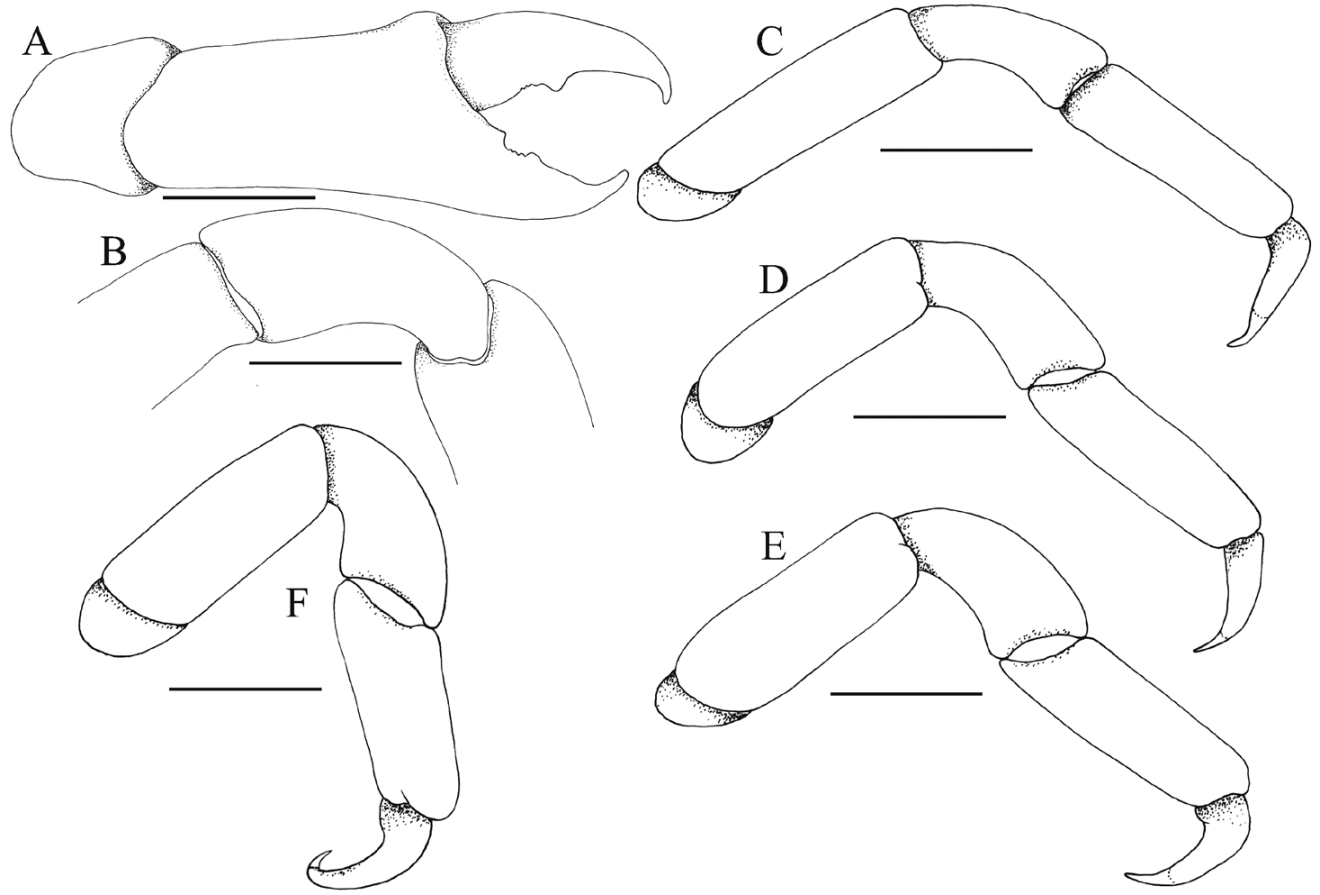
*Serenotheres janus* sp. n., holotype ♀ (8.9 × 7.9 mm) (USNM). **A** outer view of right chela **B** dorsal view of carpus of right cheliped **C–F** right P2–P5, respectively. All structures denuded. Scales = 1.0 mm.

Figs 1 , 2 , 3 , 4 , 5 , 6

##### Type material.

Holotype ♀ (8.9 × 7.9 mm) (USNM 1421642), in *Leiosolenus obesus* (Philippi, 1847) (Mollusca: Bivalvia: Mytilidae: Lithophaginae), from Njari Island, New Georgia, Solomon Islands, station SOLOM_026; 8.01374°S, 156.75649°E, coll. C. Meyer, 9 October, 2014.

##### Diagnosis.

Carapace distinctly pentagonal; lateral margins of dorsal carapace lamellum distinctly produced with posterolateral part deeply concave, highest at median cleft with 2 halves sloping gently outwards in direct frontal view; rostrum distinct with surface above antennular fossa prominently concave; surfaces of anterolateral margin and hepatic region less prominently pitted, eroded; MXP3 ischiomerus relatively more rectangular; P2 merus relatively long.

##### Description.

Carapace deep, prismatic, pentagonal in dorsal view, distinctly broader than long; anterior, lateral, and dorsal surfaces pitted (Figs 1B, 2, 3, 5A). Posterior carapace margin wide, with median part gently concave (Figs 1B, 2A, B, 5A). Frontal and antero-dorsal part of carapace raised to form distinct dorsal lamellum (Figs 1, 2, 5A). Dorsal lamellum projecting anteriorly, forming eave which overhangs true frontal margin and orbits; margins thin, glabrous; surface gently concave, regions not clearly defined, gastro-cervical grooves just visible; anterior margin acute, forming a false front, separated into 2 rounded lobes by shallow median cleft; highest at edges of median cleft, with 2 lateral halves sloping gently outwards in direct frontal view (Figs 1B, 2, 3, 4A, B). Dorsal lamellum connected to rostrum by distinct broad, longitudinal median ridge; lateral junction between dorsal lamellum and true anterolateral margin marked by distinct concavity (Figs 3, 4A, B). Surface between dorsal lamellum and true frontal and anterolateral margins deeply concave (Figs 3, 4A, B). True anterolateral margins subcristate, convex, lined with dense, short clavate setae which obscures margins (Figs 2C, 3A, 4B). True frontal margin triangular, separated into 2 low lobes by shallow concavity, lined with dense short clavate setae which completely obscures margin (Figs 3, 5B). Ventral surface of front with 2 pronounced depressions, above antennular fossae, not setose (Figs 3, 5B). Antennular fossae round, margins lined with dense short clavate setae which obscures margins; antennules folding obliquely (Figs 3B, C, 5B). Antenna with proximal 2 articles fused, immovably lodged in epistome; basal article (article 2) large, subrectangular; articles 3–5 increasingly smaller, with flagellum very short, not extending beyond orbit (Fig. 5B). Orbit small, not visible from dorsal view; cornea small, pigmented black, peduncle short; eyes just visible in frontal view (Figs 3B, C, 5B). Subhepatic region with deep, broad, slightly oblique groove, cristae delimiting groove lined with dense, short clavate setae (Figs 3, 5B). Pterygostomial region gently concave, forming shallow oblique groove (Figs 3, 5B). Epistome transversely subrectangular; median part of posterior margin gently concave (Figs 3B, 5B). Buccal cavity wide, margins lined with dense short setae (Fig. 3B, C).

MXP3 inserted obliquely, completely filling buccal cavity; outer surface and margins covered with short clavate setae which obscures structure, setae on longitudinal median part of ischiomerus distinctly less dense (Fig. 3B, C); ischium and merus completely fused without trace of suture; ischiomerus broadly subovate, longer than broad, outer surface with broad, shallow longitudinal sulcus, distal margins convex; palp 2-segmented, inserted on inner surface of ischiomerus, just below distal margin, carpus globose, dactylus conical, subspatulate, longer than carpus, extending beyond inner edge of ischiomerus; exopod elongate with basal part dilated, outer margin distinctly concave, with single elongate segment (Fig. 5C, D).

Chelipeds symmetrical, short, relatively stout, densely covered with dense short clavate setae (Figs 1B, 2C, 3); setae longer, denser on dorsal margin and along 2 longitudinal rows on outer surface of palm and carpus, forming 3 pseudo-ridges (Figs 3B, C, 4D); when denuded, surfaces of carpus and palm smooth (Fig. 6A, B). Dactylus and pollex covered with dense setae, giving fingers short, stout appearance (Figs 3B, C, 4D); when denuded, structures gently curved, tips sharp, crossing distally; cutting edge of dactylus with 1 submedian tooth and small posterior denticles; cutting edge of pollex with small tooth on proximal third, with smaller teeth and posterior denticles (Fig. 6A).

P2–5 (ambulatory legs) symmetrical from left to right, generally similar in form; margins of carpus and propodus lined with dense, long setae, setation on merus less distinct and more spare; dactylus covered with sparse long setae (Figs 1B, 2, 4C, D); relative lengths: P2>P3=P4>P5 (Fig. 6C–F). Outer surface of merus, carpus and propodus smooth when denuded; merus subovate in cross-section, unarmed (Fig. 6C–F). P2–P4 dactylus falcate, long, distinctly shorter than propodus, tip gently curved (Fig. 6C–E); P5 dactylus with distal part prominently curved, with tip hooked obliquely inwards (Fig. 6F).

Abdomen very broad, extending beyond margins of thoracic sternum, partially covering bases of P1–P4, reaching bases of MXP3 (Figs 1C, 4D); somites 1–6 and telson free; somite 4 broadest; telson broadly triangular with rounded tip (Figs 4C, D, 5E); margins lined with dense short setae which obscures margins; surface pitted, appears eroded; surface of somites 4–6 and telson concave, pits and depression on surface more prominent, with margins and edges of sutures of these somites cristate (Figs 4C, D, 5E).

##### Colour.

In life, the species is cream-yellow overall (Fig. 1).

##### Etymology.

The species is named after Janus, the ancient two-faced Roman god, alluding to the unusual two parts of the carapace when viewed dorsally. The name is used as a noun in apposition.

##### Remarks.

*Serenotheres janus* sp. n. can be separated from *Serenotheres besutensis* (Serène, 1967) in having the lateral margins of the dorsal carapace lamellum distinctly produced laterally to form a blunt angular lobe, with the posterolateral margin deeply concave (Figs 2, 5A) (vs. lateral margin not produced laterally and more rounded, with the posterolateral margin gently sinuous to almost straight in *Serenotheres besutensis*, cf. Serène 1967: pl. 2A; Ahyong and Ng 2005: fig. 5A); the dorsal carapace lamellum is highest at the median cleft, with the two halves sloping gently outwards in direct frontal view (albeit with the margins of the cleft curving downwards) (Fig. 3) (vs. dorsal carapace lamellum is lowest at the median cleft, with the two halves sloping gently inwards in direct frontal view in *Serenotheres besutensis*, cf. Serène 1967: pl. 2B; Ahyong and Ng 2005: fig. 5E); the rostrum is more prominent with the surface above the antennular fossa prominently concave (Figs 3B, C, 5B) (vs. rostrum relatively shorter with the region above the antennular fossa more shallow in *Serenotheres besutensis*, cf. Ahyong and Ng 2005: fig. 5A, C, D, E); the surface of the actual anterolateral margin and hepatic region is less prominently pitted and eroded (Figs 3, 5B) (vs. prominently pitted and eroded in *Serenotheres besutensis*, cf. Serène, 1967: pl. 2B; Ahyong and Ng 2005: fig. 5C, D); the MXP3 ischiomerus is relatively more rectangular in form (Fig. 5C, D) (vs. longitudinally ovate in *Serenotheres besutensis*, cf. Serène 1967: fig. 5; Ahyong and Ng 2005: fig. 5K); and the P2 merus is relatively longer (Fig. 6C) (vs. relatively shorter in *Serenotheres besutensis*, cf. Ahyong and Ng 2005: fig. 5G).

The type of *Serenotheres besutensis* (9.0 × 7.0 mm) (cf. Ng and Ahyong 2005) is similar to that of the holotype of *Serenotheres janus* sp. n. (8.9 × 7.9 mm), so the differences observed cannot be explained by size.

The DNA barcode sequence data of *Serenotheres janus* sp. n. indicates a novel lineage among available Pinnotheridae sequences. The closest matches are 86–85% in sequence similarity to a handful of other pinnotherid genera including *Zaops*, *Calyptraeotheres*, *Austinotheres*, and *Pinnixa* (see Palacios-Theil et al. 2009; Palacios Theil et al. 2016). To date, no other closely related species has been sequenced.

Lithophagine mussels bore into coral rock and until recently, only one species of pinnotherid crab has been reported: *Serenotheres besutensis* from an unidentified species of *Lithophaga* collected in live coral from an island off the northeast coast of Peninsular Malaysia (Serène 1967). *Serenotheres janus* sp. n. was collected from inside a large specimen of *Leiosolenus obesus* (Philippi, 1847) (Fig. 1A). The function of the unusual plates and lamellum is not known.

## Supplementary Material

XML Treatment for Serenotheres janus

## References

[B1] Ahyong ST Ng PKL (2005) Review of *Durckheimia* and *Xanthasia*, with descriptions of two new genera (Decapoda: Brachyura: Pinnotheridae). Journal of Crustacean Biology 25(1): 116–129. doi: 10.1651/C-2504

[B2] Geller J Meyer C Parker M Hawk H (2013) Redesign of PCR primers for mitochondrial cytochrome c oxidase subunit I for marine invertebrates and application in all-taxa biotic surveys. Molecular Ecology Resources 13(5): 851–861. doi: 10.1111/1755-0998.12138 23848937 10.1111/1755-0998.12138

[B3] Man JG De (1888) Report on the Podophthalmous Crustacea of the Mergui Archipelago collected by Dr. John Anderson. Journal of the Linnean Society of Zoology, London 1887, 22: 1–312, pls. 1–19.

[B4] Man JG De (1889) Über einige neue oder seltene indopacifische Brachyuren. Zoologische Jahrbücher, Abtheilung für Systematik, Geographie und Biologie der Thiere 4: 409–452, pls. 9–10.

[B5] Manning RB (1993) West African pinnotherid crabs, subfamily Pinnotherinae (Crustacea, Decapoda, Brachyura). Bulletin du Muséum national d’Histoire naturelle, Paris, series 4, 15: 125–177.

[B6] Meyer C (2003) Molecular systematics of cowries (Gastropoda: Cypraeidae) and diversification patterns in the tropics. Biological Journal of the Linnean Society 79: 401–459. doi: 10.1046/j.1095-8312.2003.00197.x

[B7] Palacios-Theil E Cuesta JA Campos E Felder DL (2009) Molecular genetic re-examination of subfamilies and polyphyly in the family Pinnotheridae (Crustacea: Decapoda). In: Martin JW Crandall KA Felder DL (Eds) Crustacean Issues 18: Decapod Crustacean Phylogenetics. CRC Press, England, 457–474. doi: 10.1201/9781420092592-c23

[B8] Palacios Theil E Cuesta JA Felder DL (2016) Molecular evidence for non-monophyly of the pinnotheroid crabs (Crustacea: Brachyura: Pinnotheroidea), warranting taxonomic reappraisal. Invertebrate Systematics 30: 1–27. doi: 10.1071/IS15023

[B9] Schmitt WL McCain JC Davidson ES (1973) Decapoda I, Brachyura I Fam. Pinnotheridae. In: Gruner H-E Holthuis LB (Eds) Crustaceorum Catalogus 3 W. Junk, Den Haag, 1–160.

[B10] Serène R (1967) Sur deux espèces nouvelles de brachyoures (Crustacés Décapodes) et sur une troisième peu connue, récoltées dans la region Malaise. Bulletin du Muséum national d’Histoire naturelle, Paris, 1966, series 2, 38: 817–827, pls. 1, 2.

[B11] White A (1846) Notes on four new genera of Crustacea. Annals and Magazine of Natural History 18: 176–178. doi: 10.1080/037454809494406

